# Supplementation of Avian Semen Extenders with Antioxidants to Improve Semen Quality—Is It an Effective Strategy?

**DOI:** 10.3390/antiox10121927

**Published:** 2021-11-30

**Authors:** Agnieszka Partyka, Wojciech Niżański

**Affiliations:** Wroclaw University of Environmental and Life Sciences, Faculty of Veterinary Medicine, Department of Reproduction and Clinic of Farm Animals, pl. Grunwaldzki 49, 50-366 Wroclaw, Poland; wojciech.nizanski@upwr.edu.pl

**Keywords:** avian semen, antioxidant, lipid peroxidation, oxidative stress, liquid storage, cryopreservation

## Abstract

Oxidative stress in sperm is a phenomenon related to the increasing rate of oxidation of cellular components and the excessive production of reactive oxygen species (ROS). The high content of polyunsaturated fatty acids in bird sperm cell membranes renders these cells particularly susceptible to lipid peroxidation (LPO). Therefore, to ensure the proper functioning of cells, it is necessary to have a balance between the formation of ROS and the protective action of the antioxidant system. This review aims firstly to briefly introduce the antioxidant system characteristics of avian semen. Secondly, we summarize the recent knowledge regarding progress in extender supplementation using antioxidants and other compounds to improve avian semen quality parameters and fertility rates. The review focuses on enzymes, vitamins, amino acids, proteins, some plant extracts, and other compounds that can be used to supplement the extenders to reduce the formation of oxidants in poultry semen and maintain its quality and enhance its fertility.

## 1. Introduction

Oxidative stress in sperm is a phenomenon related to the increasing rate of oxidation of cellular components and the excessive production of reactive oxygen species (ROS). Low levels of oxidative stress may have a beneficial effect on cells, while high levels can destroy nucleic acids, proteins, fats, and carbohydrates, ultimately leading to cell death. The influence of oxidative stress on sperm is particularly important during semen storage and semen cryopreservation because of an increased production of ROS during these processes [1]. The main sites of ROS formation are the mitochondria [2] and the sperm cell membrane [3], which are particularly vulnerable to damage resulting from rapid temperature changes.

The high content of polyunsaturated fatty acids, mainly arachidonic (20:4n-6) and docosatetraenoic (22:4n-6) acids, in the phospholipids of bird sperm cell membranes renders these cells particularly susceptible to lipid peroxidation (LPO) [4,5,6,7]. Peroxidative damage affects sperm morphology and reduces sperm motility [8], and so is believed to be the main cause of the loss of fertilizing capacity observed during the storage of bird semen [9,10].

As a result, an effective antioxidant system is essential for the protection of sperm membranes against peroxidative damage. Therefore, to ensure the proper functioning of cells, it is necessary to have a balance between the formation of ROS and the protective action of the antioxidant system.

## 2. Antioxidant System Characteristics of Avian Semen

The main function of an antioxidant defense system is to protect cells against structural damage by removing reactive oxygen metabolites. An efficient antioxidant defense system operates on three levels: preventing ROS from reacting with cell components; breaking free radical chain reactions; and removing the products of ROS reactions with cellular macromolecules [11].

The antioxidant system includes numerous antioxidants that interact with each other, and a deficiency of any can reduce total antioxidant potential. The primary enzyme antioxidant system in bird semen includes superoxide dismutase (SOD), glutathione peroxidase (GPx), and catalase (CAT).

SOD catalyzes the reduction reaction of the superoxide anion radical:

O_2_^•−^+ O_2_^•−^ + 2H^+^ → SOD → H_2_O_2_ + O_2_

Mammals and birds have three types of localized SOD proteins that differ based on the metal present in the active site (cited by [12]). Cytoplasmic SOD contains copper and zinc (Cu, Zn SOD) and is a homodimer, with each 16kDa subunit containing 151 amino acid residues. Superoxide dismutase in the mitochondrial matrix has manganese in the active center (MnSOD) and is a tetrameter with a molecular weight of about 20 kDa in each unit. Expression of MnSOD is considered essential for survival in an aerobic environment and cellular resistance to oxygen radicals. Extracellular SOD (EC-SOD) is associated mainly with polysaccharides on the cell surface and is made up of four subunits containing sugar residues. EC-SOD is structurally similar to MnSOD, but with Cu and Zn at the catalytic center.

The action of SOD is important for both the protection of the spermatogenic process and the protection of cells against O_2_^•−^ generated in the external environment. In a comparison of SOD activity in boar, rabbit, stallion, donkey, ram, bull, rooster, and human semen, donkeys were found to have the highest, and roosters the lowest, SOD activity in sperm [6,12]. Birds also have species-specific differences in SOD activity (Table 1). Turkey sperm have been shown to have lower SOD activity than rooster sperm [13], while goose sperm had higher levels of SOD than chicken [14] and turkey sperm [15]. There are also species-specific differences in SOD activity in seminal plasma, with values in water fowl found to be lower than in turkeys [15]. Further, SOD distribution is not consistent between spermatozoa and seminal plasma, with more SOD found in cells than in seminal plasma in chickens and turkeys [13].

The GPx protein is a tetramer composed of four 21.5–23 kDa subunits, each of which contains a selenium atom. This enzyme catalyzes the reduction of H_2_O_2_, as well as organic peroxides, especially lipid peroxides with reduced glutathione (GSH):

2GSH + H_2_O_2_ → GSSG + 2H_2_O

Glutathione disulfide (GSSG), which is formed in this reaction, is dangerous to the cell. It inactivates proteins, forming mixed disulfides with proteins containing thiol groups or oxidizes the thiol groups of proteins, leading to the formation of disulfide bridges. The reduction of GSSG to glutathione is catalyzed by PGx and glutathione reductase in the presence of nicotinamide adenine dinucleotide (NADPH):

GSSG + NADPH + H+ → 2GSH + NADP^+^

Glutathione peroxidase targets the attack of H_2_O_2_ on glutathione, and oxidized glutathione is reduced by glutathione reductase. Glutathione peroxidase is present in bird sperm and seminal plasma [14,15], but shows species-specific differences in activity and distribution. For example, in seminal plasma, total GPx activity (Se-dependent and non-Se-dependent) is the highest in turkey and lowest in ducks and geese. In contrast, the highest activity of this enzyme was found in geese and ducks, with much lower activity in guinea fowl [15] (Table 2). We have shown that goose semen is characterized by higher concentrations of GPx in both seminal plasma and sperm than is observed in chicken semen [14]. Moreover, we observed a negative relationship between activity of SOD and GPx in goose seminal plasma, which could explain why in ganders, only the seminal plasma SOD activity had lower levels than the other enzymes [14]. Both Se-dependent and non-Se-dependent forms of GPx have been detected in chicken seminal plasma and spermatozoa [6]. Se-GPx was found to be the main form of the enzyme in spermatozoa from such species as goose, duck, chicken, turkey, and guinea fowl. In fact, in the goose, Se-GPx was in the highest proportion, while in guinea fowl non-Se-GPx was the main form of the enzyme [15]. In mammals, eight forms of GPx have been characterized, five of which are Se-dependent (cited by [19]).

The next important enzyme is CAT. Its basic function in cells is to participate in the hydrogen peroxide disproportionation reaction:

2 H_2_O_2_ → 2 H_2_O + O_2_

The catalase protein is a tetrameter composed of four subunits with a molecular weight of approximately 60 kDa each. It contains a heme group and a binding site for NADPH. It is found in the seminal plasma and inside the sperm cells.

This enzyme works effectively in cells, protecting them against the toxic effects of hydrogen peroxide. It has two catalytic functions, depending on the concentration of H_2_O_2_. At high concentrations of H_2_O_2_, catalase acts to transform it into H_2_O and O_2_. On the other hand, when the concentration of hydrogen peroxide is low and appropriate hydrogen donors are present (e.g., ethanol, methanol), this enzyme shows peroxidase activity and removes H_2_O_2_, causing oxidation of these compounds [20].

Studies conducted on various mammalian species have shown that rabbit semen is characterized by higher CAT activity compared to bull, boar, or ram semen [21]. Until recently, there was a lack of information about the presence of CAT in bird semen, but we now know that there are differences in CAT activity between chicken and waterfowl semen [14]. In fresh chicken semen, CAT activity is lower than in goose semen, especially in sperm cells. Similarly, goose seminal plasma has a greater content of CAT than rooster semen [14] (Table 3). More recent work has shown differences in the level of CAT activity between and within exotic and indigenous Indian chicken breeds [17]. It was concluded that the high levels of LPO and greater activity of antioxidant enzymes in the roosters of these breeds indicated that the spermatozoa were under more oxidative stress, and that functionally and structurally impaired spermatozoa did not utilize these enzymes for their protection [17].

The action of antioxidant enzymes in bird semen is also supported by natural antioxidants such as vitamin E (VE), vitamin C (VC), and glutathione. Vitamin E plays a major role in the protective system of avian semen, but more importantly it is a stabilizer of sperm cell membranes [7,23]. It is mostly present in avian spermatozoa, with a very small proportion in seminal plasma [6]. Its concentration is species-specific, with higher levels in chicken than duck semen [6,7]. The VE concentration in avian spermatozoa depends on dietary supplementation [23]. 

Ascorbic acid (VC) is the second most important antioxidant playing a significant metabolic role in domestic fowl. In the chicken, VC is almost equally distributed between spermatozoa and seminal plasma [6]. These birds have the ability to synthesize vitamin C, but this ability is reduced under stress conditions. This small molecule antioxidant is present in chicken semen at 100 times the concentration of VE [6]. However, it is known that VE and VC have a strong synergistic effect on the reproductive system [24].

Glutathione possesses a thiol group which reacts with ROS and reduces H_2_O_2_ and hydroperoxides [25]. In the chicken, it is mostly found in the spermatozoa rather than the seminal plasma, and it is unlikely that it provides a major defence in antioxidant activity. It is reported that the concentration of glutathione is 2.5 times less than VC in fowl semen [6].

Breque et al. [26] proposes the following model for the organization of antioxidant protection in bird semen:

Superoxide dismutase together with GPx and proteins associated with metals constitute the first line of defense, responsible for protection against the formation of free radicals;

Natural antioxidants (vitamin A, C, E, uric acid, glutathione, and carotenoids) in combination with GPx, constitute the second level of defense, preventing and limiting the production and spread of peroxides;

The combination of numerous enzymes such as phospholipases, proteases, transferases create the third level of defense, responsible for the repair and removal of damaged particles in the cell membranes of bird sperm.

Now, the first point can be amended to include that, especially with overexpression of SOD releasing large amounts of H_2_O_2_, CAT contributes to the protection of avian sperm during oxidative stress.

## 3. Changes in Antioxidant Systems during Semen Manipulation (Dilution, Storage, Cryopreservation)

All handling procedures related to semen preparation for storage, such as dilution, cooling, freezing, exposure to cryoprotectants, and thawing, induce a higher concentration of malondialdehyde (MDA) and evoke osmotic stress which leads to oxidative stress in the semen. 

This effect is partially related to the initial extension of the semen, as seminal plasma has been shown to inhibit spontaneous lipid peroxidation. Interestingly, turkey seminal plasma has higher free radical trapping activity than chicken seminal plasma [27]. The analysis of cryopreserved semen of many mammalian species showed that during cryopreservation, the production of ROS is increased [25,28], and this has been confirmed in avian semen.

In the turkey, the total antioxidant activity was found to decline significantly following liquid storage for 72 h, with a simultaneous increase in the MDA level [18]. Even a shorter period of storage, 24 h, can cause a threefold increase in MDA in turkey semen [8]. Moreover, during storage of turkey semen in the liquid state at 4 °C for 48 h, spermatozoa lose free cholesterol and phospholipids, as the most important intracellular substrates for the respiration activity [10]. Phospholipids are lost also in chicken sperm storage over 48 h at 4 °C, due to peroxidation [29]. 

Chicken semen cryopreservation significantly increased the concentration of the final product of lipid peroxidation, MDA, in both seminal plasma and sperm cells [30]. For spermatozoa, peroxidation of lipids has critical consequences. Oxidation reactions in plasma membranes lead to the amplification of reactive oxygen species (ROS), changes in membrane fluidity, loss of compartmentalization and plasma-membrane integrity, disturbance of ion-gradients, impairment of lipid-protein interactions, and modification of DNA and proteins [31]. 

The use of C_11_-BODIPY^581/591^ indicated that the cryopreservation of fowl semen enhanced LPO in a subpopulation of live spermatozoa, compared to goose semen. Therefore it can be accepted that frozen-thawed fowl semen is more susceptible to LPO and its antioxidant system may be weaker as compared with fresh semen [32]. The freeze-thaw process also increased the concentration of MDA and reduced the total antioxidant capacity in Indian Red Jungle Fowl semen [33].

Together, these studies indicate that the natural antioxidants present in semen are not enough to protect sperm from oxidative damage during the storage process. We confirmed this assumption, showing that the activity of CAT, GPx, and SOD in chicken semen are affected by cryopreservation. Catalase appeared to play an important role in the sperm antioxidant defense strategy during cryopreservation since, unlike SOD and GPx, its content was clearly reduced, by around 14%, by the cryopreservation process [30]. We also detected decreased SOD activity in sperm cells, but with a simultaneous increase in its activity in seminal plasma. In contrast, GPx activity was higher in seminal plasma but unchanged in spermatozoa [30]. In turn, Nguyen et al. [16] reported a significant decrease in the activity of sperm SOD and GPx, but not CAT, in frozen-thawed chicken semen. 

Additionally, the capacity for stimulation of regulating kinases such as AMPK is affected after cryopreservation, with AMPK phosphorylation found to be decreased by 30% after the freeze-thaw process. However, AMPK activation plays a key role in the renewal of activity of the antioxidant enzyme system, eliminating ROS and protecting against LPO, and thereby enhancing sperm motility, viability, and other parameters of sperm metabolism and functions [16].

This phenomenon can be associated with freezability, which is lower in chicken than in goose spermatozoa [32], as is SOD activity (44.4 U/10^9^ spz vs. 308.5 U/10^9^ spz [30]), that may be connected with low sperm viability after thawing. Thus, it could be assumed that the deficiency in specialized antioxidants could not completely prevent the negative effects of LPO that occurred during prolonged in vitro storage of fowl sperm [29], and might also be insufficient to protect the gametes during cryopreservation.

## 4. Effect of Adding External Antioxidants to Diluents on Antioxidant Systems and Semen Quality during Storage and Cryopreservation

The influence of oxidative stress is particularly pronounced during semen storage and cryopreservation, with increased ROS production observed during the freezing and thawing process [28,34,35]. Spermatozoa are deprived of suitable antioxidant protection and an increase in the intensity of LPO is observed. Therefore, artificial additives for extenders used in improving mammalian semen quality are becoming increasingly popular for avian reproductive techniques, too. The selected studies on bird semen are summarized in Table 4.

### 4.1. Storage in the Liquid State

Previous studies have shown that antioxidant enzymes, as well as small molecule antioxidants could be used to improve avian semen quality during liquid storage.

Vitamin E was probably the first supplement used in an avian semen storage medium. Its addition to chicken semen stored at 4 °C improved the sperm fertilizing capacity, confirming its properties as a major chain-breaking lipid antioxidant and free radical scavenger [4]. The addition of VE to turkey semen helped to maintain sperm membrane integrity and motility during 48 h of in vitro storage [36], while in capercaillie (*Tetrao urogallus*), the addition of VE and selenium reduced the negative effects of short-term in vitro storage, increasing sperm viability and motility and protecting sperm morphology [37].

In contrast, Long and Kramer [8] found that VE supplementation of turkey sperm stored for 24 h had no beneficial effect on sperm viability and was insufficient to deter lipid peroxidation or to improve sperm motility under aerobic storage conditions.

Turkeys are of the greatest interest in the short-term storage of semen as it is the sole avian species in which reproduction takes place only through AI, using also liquid stored semen. However, turkey spermatozoa lose both phospholipids and free cholesterol during liquid storage at 4 °C for 48 h [10]. The newest research has shown that antioxidant supplementation is necessary for chilled tom semen, with the addition of glutathione and trehalose increasing total antioxidant capacity, along with individual enzymes (CAT, SOD, GPx) improving sperm plasma membrane functionality, DNA integrity, and sperm motility [18].

Another study has shown a positive impact of caffeine on some turkey sperm parameters. Caffeine affects cell metabolism, and its activity depends on the concentration of calcium ions [38]. A range of caffeine concentrations (from 0.15625 mg/mL to 7.5 mg/mL) significantly stimulated sperm motility during in vitro incubation at 5 °C. This positive effect of caffeine on motility parameters was also observed initially and after 1 h of incubation at 41 °C [39].

We also observe a constantly growing interest in the addition of different antioxidants to chicken semen stored in a liquid state. Our laboratory’s experiments have revealed a significant improvement in chicken sperm motility, progressive motility, and the proportion of rapidly motile spermatozoa, as well as an increase in kinematic parameters following the addition of 15 mM NAC and 24 h of semen storage. After 48 h, NAC and CAT maintained a significantly higher proportion of live spermatozoa [40]. 

Fattah et al. [41] attempted to enhance Beltsville Poultry Semen Extender (BPSE) using L-carnitine (LC), based on its metabolic and antioxidant roles. Antioxidant properties of LC include the scavenging of free radicals, destruction of hydrogen peroxide, and metal chelation. Moreover, it is known that this antioxidant is involved in sperm metabolism related to motility [42]. L-Carnitine also plays an important role in membrane integrity preservation, mitochondrial function, and apoptosis inhibition [43]. It is present in the semen of many species, including roosters [44]. Supplementation with 1–2 mM LC produced higher motility, viability, and membrane functionality as well as lower LPO after 24 and 48 h of storage and enhanced the fertility rate at 24 h of incubation.

Another type of antioxidant, coenzyme Q10, had beneficial effects on chicken sperm chilled with Lake extender. This oil-soluble, vitamin-like substance is present in cell membranes and lipoproteins [45]. Supplementation with 5 μM CoQ10 showed greater sperm motility and progressive motility, membrane functionality, viability, mitochondrial potential, and lower levels of LPO during 24 and 48 h of storage. Moreover, during 24 h storage, an acceptable reproductive performance was obtained when 2 and 5 μM CoQ10 was added to the extender [46].

Next, lycopene, which is the most powerful oxygen quencher of the carotenoid family, was also used for turkey semen stored at 5 °C. Addition of this antioxidant to medium protected sperm viability and decreased malonaldehyde levels for 48 h of storage [47]. This could be related to its lipophilic effect, because lycopene is accumulated in the membranes of cells and lipoproteins and creates a considerable effect within cells [48]. It seems that the efficacy of lycopene is two times more than β-carotene and ten times more than α-tocopherol [49], therefore it should be taken into consideration more often as a semen extender supplement.

### 4.2. Semen Cryopreservation

Although chicken semen is characterized by high antioxidant activity, this is not enough to completely counteract the risk of peroxidative sperm injury during semen cryopreservation [14,32,50]. Undoubtedly, cryopreservation enhances LPO [32]. Susceptibility of avian semen to LPO is caused by a reduction of antioxidant activity, causing diminished ROS scavenging, which disrupts redox homeostasis in the semen [14,30].

Many studies have indicated that diluents supplemented with antioxidants provide significant protection against ROS and the occurrence of LPO in the semen of many livestock species [25,51,52,53,54]. However, until 2013, very few of these studies were related to the cryopreservation of bird semen. It is also worth mentioning that, in most cases, previous studies were focused on the influence of dietary supplementation with antioxidants on the quality of poultry semen [26].

Starting work with antioxidant supplements in rooster semen extender, our laboratory first assessed N-acetyl-L-cysteine (NAC) and SOD as representatives of non-enzyme and enzyme antioxidants. N-acetyl-L-cysteine is a precursor for intracellular cysteine and glutathione biosynthesis and also participates in glutathione metabolism, where it acts as a stimulant of cytosolic enzymes. The sulfur-containing amino acid, cysteine, scavenges free radicals through direct chemical interactions with them [25,55]. As mentioned above, SOD is the main enzyme controlling oxidative stress in mammalian spermatozoa [56] and its activity is important for the protection of spermatogenesis and the protection of cells against O_2_^−^ produced in the external environment. Our research confirmed a significant improvement in the mitochondrial activity and motility of frozen-thawed chicken sperm following the addition of 5 mM NAC, with a similar effect obtained with the addition of 200 U/mL SOD. Both antioxidants enhanced sperm plasma membrane integrity and protected chicken sperm from apoptosis. However, only SOD supplementation significantly reduced the phenomenon of LPO in sperm cell membranes [57]. 

Similarly, Amini et al. [58] found that freezing chicken semen in a diluent supplemented with CAT (100 µg/mL) decreased LPO and consequently improved sperm motility and viability, while supplementation of the same semen extender with 50 U/mL SOD resulted in an increase in sperm motility and velocity.

Other studies focusing on CAT supplementation reported that 100 IU CAT/mL in the semen extender with 400 × 10^6^ spermatozoa resulted in a significant decrease in MDA production in rooster semen during the freeze–thawing process, with a simultaneous increase in total motility, progressive motility, viability, and membrane integrity [59,60].

Reduced glutathione, a co-factor for GPx that reacts with toxic H_2_O_2_ and hydroperoxides, was also used as an additive in chicken semen cryopreservation. It is suggested that glutathione has positive effects on mitochondrial membrane potential and fertility, as confirmed in the study of Masoudi et al. [61] that resulted in higher viability, membrane functionality, acrosomal integrity, total and progressive motility, and lower LPO.

There is also an increasing number of new antioxidants, such as Mito-TEMPO which inhibits the excessive generation of oxygen free radicals caused by freezing and thawing through the hydroxylamine-like structure of sperm cells. Tempo—piperidine nitroxide—is a SOD mimetic that functions as a SOD in the catalytic cycle, while the second compound, triphenylphosphonium (TPP+), is a lipophilic cation that accumulates several hundred fold within sperm mitochondria as a result of their membrane potential [62]. Supplementation of Lake medium with 5 and 50 μM Mito-TEMPO resulted in the amelioration of thawed rooster sperm motility, membrane functionality, mitochondria active potential, acrosome integrity, and viability, while decreasing LPO, late apoptotic-like changes, DNA fragmentation, and hydrogen peroxide content. These assessments made in vitro were confirmed by in vivo tests which reported a significant improvement in the fertilizing ability of sperm cryopreserved with TEMPO [63].

Appiah et al. [64] and Rakha et al. [65] each used quercetin, a component of flavonoids commonly found in fruits and vegetables, which has been shown to have positive effects on fresh and frozen-thawed sperm of different species [66,67]. Its properties are described as nucleophilic and it binds to DNA, providing a defense against ROS [68]. This action was demonstrated by cryopreservation of rooster semen in extender supplemented with quercetin, which resulted in reduced DFI [64] and improved chromatin condensation [65]. Additionally, the inclusion of an extender with a suitable quercetin concentration of 0.010 mg/mL emended sperm motility, viability, membrane functional integrity, acrosome integrity, and also enhanced natural antioxidant defense in the semen, simultaneously lowering MDA concentration and ROS production [64]. In Indian red jungle fowl (*Gallus gallus murghi*) roosters, 15 mM quercetin improved all of these sperm parameters [65]. 

Melatonin (ME), N-acetyl-5-methoxytryptamine, has also been tested in chicken semen extenders [69,70]. This antioxidant is known to preserve mitochondrial integrity and helps to maintain cellular function, neutralizing the toxic effects of ROS and reactive nitrogen species [71]. In our study, we found that ME decreased LPO in frozen-thawed chicken sperm and protected against DNA fragmentation. Melatonin supplementation contributed to fewer apoptotic-like changes and greater motility of thawed sperm cells [70]. Appiah et al. [69] detected a positive effect of ME on chicken sperm motility, reporting that it shielded sperm membrane integrity and acrosome integrity, augmented antioxidant activity of enzymes, and reduced oxidative stress during cryopreservation.

Lycopene has also been used in semen cryopreservation. Using encapsulation technology, Najafi et al. [72] observed a direct effect of this lipophilic carotenoid on the antioxidant status of chicken semen, enhancing the activity of GPx and TAC, and decreasing the MDA level. The addition of lycopene encapsulated in nanoliposomes resulted in both a higher fertility rate and a higher proportion of hatched eggs. The higher sperm progressive motility and membrane integrity resulting from treatment with 0.2 mM lycopene-loaded nanoliposomes likely increased the population of functional sperm in the sperm storage tubules and therefore improved fertility. This suggests that an enhanced delivery of lycopene could affect the redox balance and the energetic metabolism of the cells.

The synthesis of nanostructures and the investigation of their application in diagnosis and treatment also confirmed a positive effect of a novel formulation of ellagic acid loaded into liposomes on cryopreserved rooster sperm motility, viability, membrane functionality, and mitochondrial status, and also apoptosis [73]. Ellagic acid has antioxidant, radical scavenging, antiproliferative, and antiapoptotic properties, and significantly increased GPx and TAC after freeze-thawing. This team recently reported on the application of another novel pharmaceutical formulation, with the encapsulation of alpha-lipoic acid, an antioxidant which naturally exists in the mitochondria, in nanostructured lipid carriers (NLCs) [74]. The AI results confirmed that 30 µM alpha-lipoic acid loaded into NLCs enhanced chicken sperm fertility and was associated with a marked reduction in the percentage of apoptotic cells and with decreased active caspase-3 gene expression. Therefore, this formulation might be considered as a novel potential cryoprotectant for rooster sperm freezing.

Studies on the addition of amino acids prior to cryopreservation have also revealed their antioxidant properties. Moreover, it is known that amino acids interact with phospholipid bilayers during freezing, allowing stabilization of the cell membrane. They also reduce the concentration of toxic solutes to below the limit of toxicity, and some protect the cells against the denaturing effects of hyperosmolality during freezing [75]. Thananurak et al. [76] reported that the addition of ergothioneine, cysteamine, and serine effectively decreased malondialdehyde production in thawed chicken semen. However, only serine showed a clear positive effect on fertility.

Other amino acids, such as L-carnitine (described above), taurine, and hypotaurine have been used in chicken semen cryopreservation [77]. Hypotaurine (2-aminoethanesulfinic acid) is known to be a precursor of taurine (2-aminoethanesulfonic acid) and is the main end-product of cysteine metabolism [78]. These amino acids possess protective properties against LPO [79]. Moreover, both play an important role in DNA protection and reduce the incidence of DNA fragmentation initiated by oxidative stress [80]. The addition of L-carnitine and taurine prior to chicken semen cryopreservation was effective in protecting the sperm against apoptosis, loss of mitochondrial activity, and DNA fragmentation. Taurine supplementation was also associated with the best sperm motility. Moreover, along with hypotaurine, they suppressed the process of lipid peroxidation in cryopreserved semen, which confirmed their significant role in scavenging free radicals [77].

## 5. Conclusions

In this review, we have presented the current knowledge and the newest effective strategies exploiting antioxidants used in poultry assisted reproductive techniques. This research area is still being developed and so some aspects are not as well recognized as they are in mammals.

It is known that various factors such as genetics, physiological status, age, and management influence the antioxidant status of the mammalian ejaculate [81]. In birds, however, we still know little about the differences between species or breeds. Nevertheless, seeing the growing interest and intensity of research into the antioxidant system and its protective action against oxidative stress of various origins in bird semen, we are quite sure that more details will be available soon. In our opinion, the reader should be allowed to choose the best additive to avian semen extender, as most of the ones we have discussed here had many positive effects on avian sperm.

Although antioxidant supplementation of semen extenders has been used for many years, the supplements are now more likely to be natural substances and small particle antioxidants. Furthermore, the combination of antioxidants with nanotechnology, to maximize therapeutic activity and minimize undesirable side effects, is starting to be suggested more widely. Encapsulation of some substances which are not/weakly water soluble or with poor absorption into nanostructured lipid carriers and liposomes are promising options in active substance delivery, which might be fabricated by combining any biocompatible substances [74]. Could this perhaps be the future method for the use of different antioxidants in avian sperm preservation?

## Figures and Tables

**Table 1:** Superoxide dismutase activity in seminal plasma and in avian sperm.

Species	SOD Activity (U/mL)	SOD Activity (U/10^9^ Sperm)	References
Chicken	-	1.48	[13]
	-	22.04	[15]
	46.2	4.4	[14]
	91.7	133	[16]
	-	31.36–157.96	[17]
Turkey	-	16.97	[15]
	77.71	0.32	[13]
	-	93.21	[18]
Guinea fowl	65.98	21.91	[15]
Ducks	32.27	59.91	[15]
Goose	42.05	77.74	[15]
	73.7	308.5	[14]

**Table 2:** Glutathione peroxidase activity in seminal plasma and in avian sperm.

Species	GPx Activity (U/mL)	GPx Activity (U/10^9^ spz)	References
Chicken	268.63 - -	44.46 35.5 mU/10^9^ 0.22–0.94	[15] [16] [17]
Turkey	372.20 20.9	31.75 -	[15] [18]
Guinea fowl	240.64	11.83	[15]
Duck	136.43	72.57	[15]
Goose	159.17	178.68	[15]

**Table 3:** Catalase activity in seminal plasma and in avian sperm.

Species	CAT Activity (U/mL)	CAT Activity (U/10^9^ Sperm)	References
Chicken	46.8 - - - 38.10	9.7 8.38–57.61 31 59.3 -	[14] [17] [16] [18] [22]
Goose	76.3	239.2	[14]

**Table 4:** Effects of antioxidants during semen storage on spermatozoa quality of avian species.

Antioxidant	Species	Antioxidant Concentration	Semen Storage Type	Outcomes of Antioxidant Supplementation	References
Vitamin E (VE)	Chicken	2; 4; 8; 12;16; 20; 40 µg/mL	Liquid	Does not improve motility; improves fertility rate (8 µg/mL)	[4]
	Turkey	1; 2; 5; 10; 20; 40; 80 µg/mL	Liquid	Maintains sperm membrane integrity and motility	[36]
	Turkey	10; 40 µg/mL	Liquid	Does not improve sperm viability and MDA level	[8]
Vitamin E (VE) + selenium (Se)	Capercaillie (*Tetrao urogallus)*	8 mg/mL VE; 1 mg/mL Se	Liquid	Increases sperm motility, positively affects sperm morphology	[37]
Glutathione (GSH)	Turkey	0.5; 1; 2 mM	Liquid	Increases sperm motility (1, 2 mM); enhances plasma membrane integrity, DNA integrity, total antioxidant activity and decreases LPO (2 mM)	[18]
	Chicken	0.5; 1; 2; 4; 8 mM	Cryopreservation	Increases viability, membrane functionality, mitochondrial activity, total and progressive motility, fertility rate; decreases MDA level (2, 4 mM)	[61]
Trehalose	Turkey	5; 75; 100 mM	Liquid	Improves sperm motility, DNA integrity, plasma membrane integrity and functionality, and oxidative stress (75 mM)	[18]
Caffeine	Turkey	0.07812; 0.15625; 0.3125; 0.625; 1.25; 2.5; 5; 7.5; 10 mg/mL	Liquid	Stimulates sperm motility and progressive motility (0.15625 to 7.5 mg/mL); does not improve sperm viability	[39]
N-acetyl-L-cysteine (NAC)	Chicken	5; 15 mM	Liquid	Improves sperm motility, progressive motility, kinetic parameters, and viability (15 mM)	[40]
	Chicken	5 mM	Cryopreservation	Improves mitochondrial activity and sperm motility; enhances sperm plasma membrane integrity and protects against apoptosis	[57]
Catalase (CAT)	Chicken	100; 300 U/mL	Liquid	Improves sperm viability	[40]
	Chicken	50; 100; 200; 300 µg/mL	Cryopreservation	Improves sperm motility and viability, decreases MDA level (100 µg/mL)	[58]
L-carnitine (LC)	Chicken	0.5; 1; 2; 4; 8 mM	Liquid	Increases motility, viability, and membrane functionality, decreases LPO, enhances fertility rate (1, 2 mM)	[41]
	Chicken	1; 5 mM	Cryopreservation	Protects against apoptosis, LPO, and DNA fragmentation; increases mitochondrial potential (1 mM)	[77]
Coenzyme Q10 (CoQ10)	Chicken	1; 2; 5; 10 µM	Liquid	Increases sperm motility and progressive motility, membrane functionality, viability, mitochondrial potential and decreases LPO (5 µM); increases fertility rate (2, 5 µM)	[46]
Lycopene	Turkey	0.05; 0.1 mg/mL	Liquid	Protects sperm viability and decreases MDA levels	[47]
Lycopene—loaded liposomes	Chicken	0.1; 0.2; 0.3 mM	Cryopreservation	Increases sperm motility and progressive motility, viability, membrane functionality, mitochondrial activity, GPx activity, and TAC; decreases MDA level and apoptosis; enhances fertilizing rate (0.2 mM)	[72]
Superoxide dismutase (SOD)	Chicken	200 U/ml	Cryopreservation	Reduces LPO in sperm cell membranes; enhances sperm motility, plasma membrane integrity, and protects sperm against apoptosis	[57]
	Chicken	50; 100; 200; 300 U/mL	Cryopreservation	Increases sperm motility and viability (50 U/mL)	[58]
Mito-TEMPO	Chicken	0.5; 5; 50; 500 μM	Cryopreservation	Increases sperm motility, membrane functionality, mitochondrial potential, acrosome integrity and viability, decreases LPO, late apoptotic-like changes, DNA fragmentation and hydrogen peroxide content; improves fertility rate (5, 50 μM)	[63]
Quercetin	Chicken	0.005; 0.01; 0.02; 0.04 mg/mL	Cryopreservation	Increases sperm motility, membrane and acrosome integrity, mitochondrial activity, viability, protects morphology, increases GPx, SOD, CAT activity, decreases MDA level and ROS production, DNA fragmentation (0.01 mg/mL)	[64]
	Indian red jungle fowl (*Gallus gallus murghi*)	5; 10; 15; 20 mM	Cryopreservation	Improves sperm motility, plasma membrane integrity, viability, acrosome integrity, chromatin condensation, mitochondrial activity and TAC; decreases MDA level (15 mM)	[65]
Melatonin (ME)	Chicken	1 mM; 1 μM; 1 nM	Cryopreservation	Increases sperm motility, membrane integrity, mitochondrial activity; decreases LPO, apoptosis, protects against DNA fragmentation (1 mM; 1 μM)	[70]
	Chicken	0.125; 0.25; 0.5 mg/mL	Cryopreservation	Improves sperm motility, membrane and acrosome integrity, augments antioxidant activity of enzymes and reduced oxidative stress (0.25 mg/mL)	[69]
Ellagic acid—loaded liposomes	Chicken	0.5; 1; 2 mM	Cryopreservation	Increases sperm motility, progressive motility, viability, membrane functionality, mitochondrial activity, decreases apoptosis and MDA level; increases TAC and GPx, SOD activity (1 mM)	[73]
Alpha-lipoic acid loaded lipid nanoparticles	Chicken	10; 20; 30; 40; 50 μM	Cryopreservation	Increases total and progressive motility, protects membrane integrity, mitochondria activity, viability and against apoptosis; decreases MDA level, and increases GPx, SOD activity and TAC (30 μM)	[74]
Ergothioneine (ERG)	Chicken	0.01; 0.02; 0.04; 0.06; 0.08; 0.1; 0.5; 1; 5 mM	Cryopreservation	Decreases sperm motility (0.01 to 0.04 mM); decreases MDA	[76]
Cysteamine (AET)	Chicken	0.001; 0.002; 0.004; 0.006; 0.008; 0.01; 0.05; 0.1; 0.5 mM	Cryopreservation	Decreases sperm motility (0.001 and 0.004 mM); decreases MDA	[76]
Serine (SER)	Chicken	1; 2; 4; 6; 8; 10; 15; 20 mM	Cryopreservation	Increases motility sperm membrane integrity and fertility rate; decreases MDA (4 mM)	[76]
Taurine (T)	Chicken	1; 10 mM	Cryopreservation	Increases sperm motility, 1 mM protects plasma membrane integrity, against apoptosis and DNA fragmentation; reduces LPO	[77]
Hypotaurine (HT)	Chicken	1; 10 mM	Cryopreservation	Increases sperm motility; reduces LPO	[77]
